# Effectiveness and Safety of Glycopyrronium–Formoterol–Budesonide Triple Therapy in Chronic Obstructive Pulmonary Disease (AIR-FORCE): An Open-Label Multi-Centric Phase 4 Study

**DOI:** 10.3390/arm93060053

**Published:** 2025-11-25

**Authors:** Anjali R. Nath, Adesh Kumar, Amit Suresh Bhate, Bharat Mehrotra, Deependra Kumar Rai, Vijay Kumar Barge, Divya Bhojwani, Sagar Bhagat, Sumit Bhushan, Saiprasad Patil, Hanmant Barkate

**Affiliations:** 1 Citizen Hospital, Bangalore 560002, India; anjali.rnadh@gmail.com; 2 Respiratory Medicine, Uttar Pradesh University of Medical Sciences (UPUMS), Etawah 206130, India; dradeshkumar1974@gmail.com; 3 Medicine Department, Jeevan Rekha Hospital, Belagavi 590002, India; dr.amitsureshbhate@gmail.com; 4 New Leelamani Hospital, Kanpur 208001, India; bharatdoc14@gmail.com; 5 Respiratory Medicine, All India Institute of Medical Sciences (AIIMS), Patna 801507, India; drdeependrak@aiimspatna.org; 6 Medicine Department, Rajarshee Chhatrapati Shahu Maharaj Government Medical College Chhatrapati Pramila Raje Hospital (RCSM), Kolhapur 416012, India; drvijaybarge12@gmail.com; 7 Global Medical Affairs, Glenmark Pharmaceuticals, Ltd., Mumbai 400099, India; sagar.bhagat@glenmarkpharma.com (S.B.); sumit.bhushan@glenmarkpharma.com (S.B.); saiprasad.patil@glenmarkpharma.com (S.P.); hanmant.barkate@glenmarkpharma.com (H.B.)

**Keywords:** COPD, glycopyrronium/formoterol/budesonide, GFB, real-world, effectiveness, safety, metered-dose inhaler, dry-powder inhaler

## Abstract

**Highlights:**

**What are the main findings?**

- Glycopyrronium–formoterol–budesonide triple therapy improved lung function, symptoms, and adherence in Indian COPD patients in a real-world setting.
- The treatment was well tolerated, with only mild adverse events and no new safety concerns.

**What are the implications of the main findings?**

- Single-inhaler triple therapy can be significantly beneficial for COPD patients who continue to be symptomatic on dual therapy.
- Availability of both metered-dose and dry-powder inhalers supports customized treatment, enhancing adherence and patient outcomes.

**Abstract:**

Chronic obstructive pulmonary disease (COPD) is a major health burden in India with limited real-world data on triple inhaler therapy. This prospective, open-label, multi-center, single-arm, phase 4 study (October 2023–August 2024) assessed the effectiveness and safety of glycopyrronium/formoterol fumarate/budesonide (GFB) triple therapy, administered as metered-dose inhaler (MDI) or dry-powder inhaler (DPI), in Indian COPD patients. Symptomatic patients aged ≥40 years with minimum one exacerbation in the past year and receiving dual or monotherapy were included. GFB was delivered as MDI or DPI based on physician and patient preference. Primary outcomes were changes from baseline in trough forced expiratory volume in 1 s (FEV_1_), forced vital capacity (FVC), and modified medical research council (mMRC) score over 24 weeks, with assessment of exacerbations, hospitalizations, rescue medication use, and safety. In 184 patients (70.65% male, mean age 53.7 years), GFB significantly improved FEV_1_, FVC, and mMRC scores. Eleven mild exacerbations were reported without hospitalization; 17.39% used rescue salbutamol largely in the first 4 weeks. GFB was well tolerated, with mild-to-moderate adverse events in 14.67%, and outcomes were comparable between MDI and DPI. Our findings support GFB as safe and effective treatment in real-world COPD management.

## 1. Introduction

Chronic obstructive pulmonary disease (COPD) is a complex and progressive inflammatory lung disorder accounting for around 6% of global deaths [1]. In 2021, COPD affected an estimated 213.39 million people and was responsible for 3.72 million deaths globally [2]. The prevalence of, and deaths due to, COPD are higher in East Asia and South Asia compared to other parts of the world [2]. In India, COPD poses a significant public health challenge with the estimated prevalence among Indian adults at 7.4%, with higher rates observed in urban areas and among males [3]. This translates to approximately 37.6 million individuals affected by COPD across India [4].

Treatment objectives of COPD, as outlined by the Global Initiative for Obstructive Lung Disease (GOLD), are symptom control, reduction in the frequency and severity of exacerbations, as well as improvement in the health-related quality of life (HRQoL) and exercise tolerance [1]. Bronchodilators are the mainstay in COPD management, and combining bronchodilators that act by different mechanisms, including long-acting muscarinic antagonists (LAMAs) such as glycopyrronium and long-acting beta-2 agonists (LABAs) such as formoterol, can result in better treatment outcomes and prevention of exacerbations compared to monotherapy [1,5,6,7]. Despite treatment with dual bronchodilation (LABA/LAMA), many patients remain symptomatic and continue to experience exacerbations, highlighting the need for escalation of therapy. Adding inhaled corticosteroids (ICS) such as budesonide to the LABA/LAMA combination as a step-up triple therapy in patients with moderate to severe COPD has been demonstrated to reduce exacerbations, improve lung function, and enhance patient-reported outcomes compared to LABA monotherapy, LAMA monotherapy, or dual therapies with LABA/LAMA or LABA/ICS [1,8,9,10]. Further, patient adherence to medication has been reported to be higher with triple therapy administered in a single inhaler compared to multiple-inhaler triple therapy [11].

Glycopyrronium/formoterol fumarate/budesonide (GFB) is a fixed-dose triple combination of glycopyrronium (a LAMA), formoterol fumarate (a LABA), and budesonide (an ICS). It was approved for COPD management in India. In India, it is available as both metered-dose inhalers (MDIs: 9/4.8/160 mcg and 9/4.8/200 mcg) and dry-powder inhalers (DPI: 25/12/400 mcg); the efficacy and safety of GFB when administered via either formulation have been shown to be similar [12].

Despite these findings, there is a dearth of studies exploring the real-world effectiveness and safety of GFB in Indian patients with COPD. With this context, the present study was planned to address this gap, with the specific objective of evaluating the real-world effectiveness and safety of GFB triple therapy when given as either MDI or DPI, in managing COPD among Indian patients.

## 2. Materials and Methods

### 2.1. Study Design and Study Participants

This prospective, open-label, interventional, multi-center, single-arm, phase 4, real-world study codenamed ‘AIRFORCE’ study (CTRI: CTRI/2023/10/058675) was conducted at six research sites within India. Eligible patients were aged ≥40 years, belonged to either sex, had a documented physician diagnosis of COPD (based on the American Thoracic Society (ATS)/European Respiratory Society (ERS) [13] or GOLD 2024 [1] guidelines), had at least one exacerbation episode in the past year, were currently symptomatic and on maintenance treatment with ICS/LABA, LAMA/LABA, or LAMA, and demonstrated a capability to use MDI/DPI correctly and independently. Patients with documented current diagnosis of asthma, COPD patients currently receiving triple therapy (ICS/LABA/LAMA), and patients with any allergies or conditions that made them unsuitable for trial participation from the investigator’s viewpoint were excluded from the study.

### 2.2. Methods

Eligible patients who provided informed consent to participate in the study underwent baseline examination upon recruitment. Each eligible patient was initiated with GFB therapy in the form of either a DPI (25/12/400 mcg per capsule, respectively, one capsule twice daily), or an MDI (9/4.8/160 mcg per actuation, respectively, two inhalations twice daily) as per routine clinical practice, in accordance with the prescribing information. Selection of the mode of inhaler was based on investigator’s clinical judgement, patient preferences, and patients’ ability to use the device. SABA therapy in the form of MDI was permitted as rescue medication throughout the study; however, patients were prohibited from using any systemic or inhaled corticosteroids, systemic methylxanthines, or bronchodilators apart from the study medication throughout the study. Patients were also given diary cards to record day-to-day symptoms, adverse events (AEs), and rescue medication usage.

There were three in-clinic follow-ups (at weeks 4, 12, and 24) during which effectiveness, safety, medication adherence, and satisfaction were assessed. Two telephonic follow-ups were conducted at weeks 16 and 20 for safety and symptom assessment. Unscheduled visits based on patient’s request or physician’s necessity were permitted throughout the study.

### 2.3. Outcomes and Their Measurement

The primary effectiveness outcomes were changes from baseline in the trough forced expiratory volume in 1 s (FEV_1_) and modified medical research council (mMRC) score at 24 weeks. Additional effectiveness outcomes included mean change in forced vital capacity (FVC), number of patients with COPD exacerbations, number of patients requiring COPD-related hospitalization, and rescue medication requirement over 24 weeks. Patients’ adherence to the medication, and satisfaction with the treatment of both patients and physicians were also measured. The safety outcomes included number of patients with any treatment-emergent AE (TEAE), drug-related TEAE, and serious TEAEs over 24 weeks.

### 2.4. Data Handling, Statistical Analysis, and Data Availability

All data were entered electronically, and all statistical analyses were performed using R software version 4.3.2. The following R packages were utilized for analysis: dplyr, ggplot2, stats, flextable, readr, readxl, and tidyr. The Wilcoxon signed-rank test was applied for analysis. Continuous variables were summarized using descriptive statistics, including the number and proportion of observations, mean, and standard deviation (SD). Effectiveness analyses were performed on the full analysis set (which included all enrolled patients), and safety analyses were performed on the safety set (which included all enrolled patients who received at least one dose of GFB). Comparisons of continuous variables employed the student’s *t*-test, while skewed distributions were analyzed using the Wilcoxon signed rank test for paired data, with adjusted *p*-values. A *p*-value of <0.05 was considered statistically significant. Categorical data were analyzed as proportions. The sample size of 184 participants was determined using PASS 2022 (v22.0.2), assuming a 17.5% incidence of drug-related TEAEs to estimate this proportion with a two-sided 95% confidence interval of maximum width 0.11.

### 2.5. Ethics Committee and Informed Consents

Approval was obtained for the study protocol from the ethics committee of each participating center (additional details in Supplementary Table S1), and written informed consent was collected from all eligible participants before initiating any study procedure. The study was conducted in complete adherence with the principles enshrined in the Declaration of Helsinki and Good Clinical Practice.

## 3. Results

### 3.1. Demographics and Baseline Characteristics

Between October 2023 and August 2024, 184 patients were enrolled in the study, with 92 patients each receiving GFB in DPI and MDI formulations. Two patients withdrew consent during the study after initiation of study medication. Thus, while 182 patients completed the study, all 184 patients were included in the full analysis as well as the safety analysis sets (Supplementary Figure S1). The baseline and demographic details of the patients are summarized in Table 1. The baseline parameters were similar between patients who received DPI and MDI formulations of GFB.

### 3.2. Effectiveness Outcomes

#### 3.2.1. Improvement in Lung Function and Symptoms

**Figure 1:**
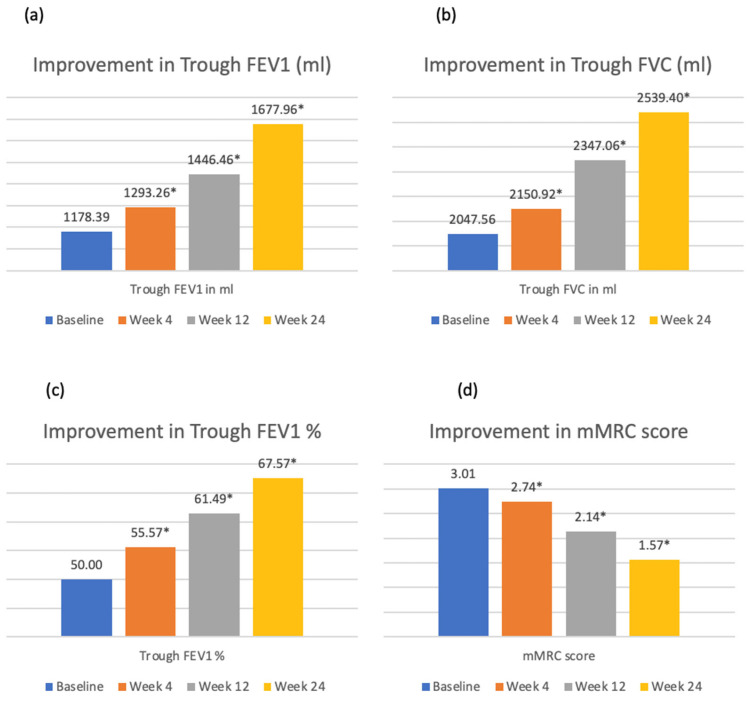
Improvements in (**a**) trough FEV_1_ (mL), (**b**) trough FVC (mL), (**c**) trough FEV_1_%, and (**d**) mMRC score over 24 weeks of therapy with GFB (glycopyrronium/formoterol fumarate/budesonide) triple therapy. Note: * *p* < 0.05 compared to baseline. FEV_1_ = forced expiratory volume in 1 s; FVC = forced vital capacity; mMRC = modified medical research council.

After 24 weeks of therapy, GFB was associated with statistically significant improvements in trough FEV_1_, trough FVC, as well as mMRC scores compared to baseline (Table 2, Figure 1). A similar improvement was seen with both DPI and MDI formulations of GFB (Supplementary Table S2).

#### 3.2.2. Exacerbation Reduction and Rescue Medication Use

At week 4, 5 patients (2.72%) experienced mild exacerbations, followed by 4 (2.17%) by week 12 and 3 (1.65%) by week 24. Notably, no patients experienced moderate or severe exacerbations throughout the study period.

With regards to rescue medications, a total of 32 (17.39%) patients reported rescue salbutamol use during the study period, of which most [15 (46.88%)] were in the first 4 weeks after starting GFB therapy. In the subsequent weeks, the rescue medication usage was lower, with 11 (34.38%) patients and 6 (18.8%) patients reporting salbutamol usage in weeks 4–12 and 12–24, respectively.

The trend was similar with both DPI and MDI formulations of GFB over the weeks (Supplementary Tables S3 and S4).

#### 3.2.3. Compliance with Study Medication

Compliance to GFB therapy remained consistently high throughout the 24-week treatment period. At weeks 4, 12, and 24, all subjects demonstrated 100% compliance with the study medication. This trend was observed consistently across both DPI and MDI user groups (Supplementary Table S5).

#### 3.2.4. Patient and Physician Satisfaction

**Figure 2:**
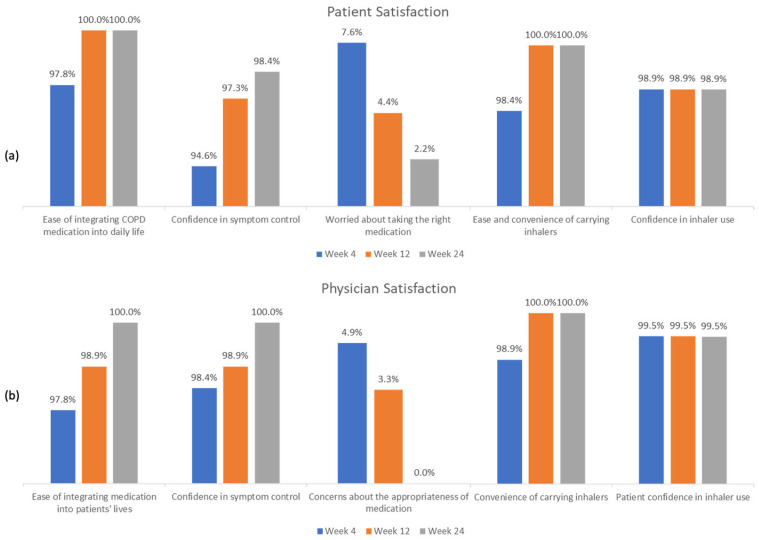
Satisfaction with GFB (glycopyrronium/formoterol fumarate/budesonide) triple therapy over 24 weeks for COPD: (**a**) patient and (**b**) physician perspectives.

Patient and physician satisfaction remained high throughout the study, across all five domains evaluated, with similar levels of satisfaction observed for both DPI and MDI formulations (Figure 2, Supplementary Tables S6 and S7).

### 3.3. Safety Outcomes

Out of 184 patients in the safety population, a total of 27 (14.67%) patients experienced 29 TEAEs as summarized in Table 3. Most of the AEs reported were mild, 25 (86.20%), and few, 4 (13.79%), were moderate. Amongst the total population, AEs reported in 17 (9.24%) patients were considered related to the treatment while AEs in 10 (5.43%) patients were determined unrelated to treatment by an investigator. No serious AEs were reported during the study period. All TEAEs were completely resolved by the end of the study. Both DPI and MDI formulations of GFB demonstrated a similar safety profile (Supplementary Table S8).

## 4. Discussion

The AIRFORCE study provided real-world evidence demonstrating both MDI or DPI GFB inhalers were effective and well tolerated among Indian COPD patients producing clinically meaningful improvements in lung function and adherence. This is particularly important in Indian context, where the burden of COPD is significantly high, and there is a need for an effective drug and device choice.

Lung function improvement of approximately 500 mL, as evidenced by a significant increase in trough FEV_1,_ was a key finding of this study and was notably higher than what is reported in RCTs such as KRONOS [14] and ETHOS [15]. In the KRONOS study (2018), over 24 weeks, GFB MDI demonstrated significant improvements in lung function compared to dual therapies. It showed greater FEV_1_ AUC_0-4_ versus budesonide/formoterol fumarate (BFF) MDI (104 mL) and BFF DPI (91 mL), and also improved morning pre-dose trough FEV_1_ versus GFF MDI (22 mL) and BFF MDI (74 mL), with all differences being statistically significant [14].

The improvement in FEV_1_ observed in the AIRFORCE study, despite being a phase 4 trial, was notably higher compared to other single-inhaler triple therapies (SITTs) used in the management of COPD. For instance, landmark phase 3 studies like the IMPACT trial reported a trough FEV_1_ improvement of 97 mL [16], while the TRINITY study demonstrated an increase of 82 mL at 52 weeks of therapy [17]. The extent of improvement in lung function seen in AIRFORCE might represent the typical real-world COPD management, where delays in escalation to triple therapy and suboptimal inhaler use are common [18]. The organized support provided in this study—especially inhaler training, consistent follow-ups, and monitoring of adherence—may have contributed to the observed magnitude of benefit. Further studies are required to explore the impact of these real-world factors on the magnitude of results. These results emphasize that in everyday clinical settings, optimized SITT therapy can lead to significant functional recovery, particularly for patients who begin with a low baseline or have been poorly managed before.

A sub-group analysis of KRONOS study also demonstrated that GFB was effective in reducing exacerbations even among patients who had no history of exacerbations in previous year, a group that comprised nearly 70% of the study population [19]. Similarly, ETHOS [15] was associated with a significantly lower annual rates of moderate or severe COPD exacerbations (1.07 to 1.08 with GFB, compared to 1.24 with B/F and 1.42 with G/F dual therapies). GFB triple therapy significantly prolonged the time to the first moderate or severe exacerbation, and was also associated with greater improvements in symptom control and HRQoL, compared to both dual therapy regimens [15]. These effects were consistent across subgroups, regardless of prior ICS use [20]. In our study, over 24 weeks of GFB therapy, exacerbations were reported in 12 (6.52%) patients, all of which were mild. This highlights the potential role of GFB in reducing exacerbations in real-world settings thereby contributing to disease stability. These findings align with the benefits of SITT seen in pivotal trials, with annual moderate or severe exacerbation rates of 0.91 vs. 1.07 (dual therapy) in IMPACT [16] and 0.50 vs. 0.59 in TRIBUTE [9].

The impact of GFB therapy on rescue medication use was explored in both KRONOS and ETHOS. While ETHOS trial reported fewer daily puffs of rescue medication for patients on GFB compared to those on dual therapy [15], KRONOS did not observe a statistically significant difference in rescue medicine use between the groups [14]. In our study, rescue salbutamol use was highest in the first four weeks and declined progressively, suggesting good disease control over time. GFB was well tolerated in both KRONOS and ETHOS, similar to our study [14,15]. Further, a post hoc analysis of ETHOS reported lower cardiopulmonary mortality with GFB, suggesting potential additional systemic benefits [21].

Several other randomized trials have reported the beneficial effect of GFB combination therapy over monotherapy or dual therapy [9,17,22]. Our study results complement these efficacy findings from controlled settings, and demonstrate that GFB has beneficial effects on FEV_1_, FVC, exacerbations, and rescue medications use in real-world settings as well. Similar real-world benefits of GFB therapy have also been reported in retrospective studies [23,24,25,26].

Beyond effectiveness and safety outcomes, our study also evaluated satisfaction with therapy from both the physician and patient perspectives. Our study demonstrated a statistically significant reduction in the mMRC score with GFB therapy over 24 weeks, signifying a considerable reduction in the grade of dyspnea as perceived by the patients. We also observed a high degree of physician and patient satisfaction with GFB therapy, which reflects the beneficial respiratory effects of GFB among the patients.

GFB’s availability in DPI and MDI forms is a distinct advantage, allowing patients to choose based on preference and ability, thus improving adherence to treatment [11]. This was evident in our study, with medication adherence remaining high throughout the study period. In fact, all patients in our study demonstrated >80% adherence to medication. Our study did not find differences in outcomes between MDI and DPI formulations of GFB, similar to previous reports [12,27]. MDI and DPI formulations each have distinct advantages and limitations. MDIs require precise coordination between actuation and inhalation, which may be challenging for elderly patients or those with poor hand–breath coordination. DPIs, on the other hand, are breath-actuated, eliminating the need for synchronization but requiring sufficient inspiratory effort, which may be reduced in patients with severe airflow limitation [27]. The availability of GFB in both MDI and DPI formulations permits a tailored approach based on individual patient capabilities and preferences.

The safety profile in this study is in line with global data, with no new safety signals identified. AE rates were comparable to those reported in phase 3 trials, where GFB showed a favorable safety profile. Similar to the findings of ETHOS and KRONOS studies [14,15], most TEAEs in our study affected the respiratory system, with the most frequent TEAE observed being cough. All TEAEs in our study were mild or moderate in severity, demonstrating the safety of GFB therapy. The two formulations were similar with respect to safety, mirroring the findings of previous studies [12].

Our results must be interpreted in the backdrop of limitations such as the absence of a comparator arm, reliance on physician discretion for inhaler selection, and a relatively short follow-up duration. Specifically, the absence of a control or comparison group limits the interpretability of the results, making it challenging to attribute observed improvements solely to GFB therapy. Further, our study did not adjust for confounders such as duration of prior dual therapy, socioeconomic factors, or adherence behavior outside study monitoring, since this was a phase 4, real-world study. Although the open-label design and investigator discretion in inhaler selection could potentially introduce bias, the impact of these factors was minimized through investigator training, standardized assessment procedures, and objective outcome measurements. Despite these limitations, our study has several strengths, including its multi-centric design, real-world patient cohort, and comprehensive assessment of both efficacy and safety outcomes. Future research should focus on long-term outcomes, comparative assessment with other triple therapy options for COPD, cost-effectiveness of therapy, and healthcare resource utilization patterns to further define the role of GFB in COPD management.

## 5. Conclusions

To conclude, consistent with global clinical studies, our study demonstrated sustained improvement in lung function, reduction in exacerbations, and overall improved respiratory outcomes with GFB irrespective of MDI or DPI formulations, among Indian COPD patients who had symptoms despite being on dual therapies. GFB also reduced rescue medication use, and was well tolerated with no new safety signals in a real-world setting, supporting findings from controlled clinical studies. Our findings further strengthen the available clinical evidence of GFB triple therapy as an effective and safe therapeutic option in the management of COPD among Indian patients.

## Figures and Tables

**Table 1:** Baseline parameters.

Parameter	All Patients Enrolled (N = 184)
Age, mean ± SD	53.71 ± 9.77
Gender, n (%)	
Male	130 (70.65%)
Female	54 (29.34%)
Baseline effectiveness parameters	
FEV_1_ (mL), mean ± SD	1178.39 ± 369.89
FEV_1_% predicted, mean ± SD	50.00 ± 11.41
mMRC score, mean ± SD	3.01 ± 0.66
Comorbidities	
Hypertension, n (%)	30 (18.68%)
Diabetes mellitus, n (%)	16 (8.69%)
Cardiovascular disorder, n (%)	5 (2.72%)
Others, n (%)	2 (1.08%)
Mean number of episodes of exacerbation in past 1 year	1.22 ± 0.41
Prior medication	
Any ICS + any LABA, n (%)	120 (65.21%)
Any LABA + any LAMA, n (%)	64 (34.78%)

Note: FEV_1_ = forced expiratory volume in 1 s; mMRC = modified medical research council; ICS = inhaled corticosteroid; LABA = long-acting beta agonist; LAMA = long-acting muscarinic antagonist.

**Table 2:** Effectiveness after 24 weeks of GFB (glycopyrronium/formoterol fumarate/budesonide) triple therapy compared to baseline.

Parameter	Baseline (Mean ± SD)	24 Weeks (Mean ± SD)	Change from Baseline to 24 Weeks (Mean ± SD)	*p* Value vs. Baseline
Trough FEV_1_ in mL	1178.39 ± 369.89	1677.96 ± 500.54	499.57 ± 130.65	<0.001
Trough FEV_1_%	50.00 ± 11.41	67.57 ± 8.91	17.57 ± 2.5	<0.001
Trough FVC in mL	2047.56 ± 547.92	2539.40 ± 640.79	491.84 ± 92.87	<0.001
mMRC score	3.01 ± 0.66	1.57 ± 0.75	−1.44 ± 0.09	<0.001

Note: FEV_1_ = forced expiratory volume in 1 s; FVC = forced vital capacity; mMRC = modified medical research council.

**Table 3:** Safety outcomes of 24 weeks of GFB therapy for COPD management.

Adverse Event	Number of Adverse Events Reported, n (%)
All TEAEs	29 (15.76%)
Respiratory, thoracic, and mediastinal disorders	
Cough	10 (5.43%)
URTI	4 (2.17%)
Cold	1 (0.54%)
Infections and Infestations	
UTI	2 (1.09%)
Musculoskeletal and Connective Tissue disorder	
Muscle spasm	2 (1.09%)
Back pain	1 (0.54%)
Nervous System Disorder	
Headache	2 (1.09%)
General disorder and administration site conditions	
Fever	2 (1.09%)
Dry mouth	1 (0.54%)
Diarrhea	1 (0.54%)
Nausea	1 (0.54%)
Heartburn	1 (0.54%)
Renal and Urinary Disorders	
Burning micturition	1 (0.54%)

Note: COPD = chronic obstructive pulmonary disease; TEAE = treatment-emergent adverse event; URTI = upper respiratory tract infections; UTI = urinary tract infections.

## Data Availability

The original contributions presented in this study are included in the article/Supplementary Material. Further inquiries can be directed to the corresponding author(s).

## Supplementary Materials

The following supporting information can be downloaded at https://www.mdpi.com/article/10.3390/arm93060053/s1; Figure S1: Flowchart for patient selection (CONSORT 2025); Table S1: Details of ethics committees and approvals; Table S2: Effectiveness after 24 weeks of GFB (glycopyrronium/formoterol fumarate/budesonide) triple therapy given as dry-powder inhaler (DPI) or metered-dose inhaler (MDI) formulation, and overall effectiveness, compared to baseline; Table S3: Number of subjects with exacerbations (mild/moderate/severe) over 24 weeks; Table S4: Rescue medication use over 24 weeks of therapy with GFB; Table S5: Compliance with study medication over 24 weeks of therapy; Table S6: Patient satisfaction with GFB treatment over 24 weeks for GFB-MDI, GFB-DPI, and overall groups; Table S7: Physician satisfaction with GFB treatment over 24 weeks for GFB-MDI, GFB-DPI, and overall groups; Table S8: Detailed group-wise safety outcomes of 24 weeks of GFB therapy for COPD management.

## Abbreviations

The following abbreviations are used in this manuscript:

COPD	Chronic obstructive pulmonary disease
GOLD	Global Initiative for Obstructive Lung Disease
HRQoL	Health-related quality of life
LAMAs	Long-acting muscarinic antagonists
LABAs	Long-acting beta-2 agonists
ICS	Inhaled corticosteroids
GFB	Glycopyrronium/formoterol fumarate/budesonide
MDI	Metered-dose inhaler
DPI	Dry-powder inhaler
ATS	American Thoracic Society
ERS	European Respiratory Society
mMRC	Modified medical research council
FVC	Forced vital capacity
TEAE	Treatment-emergent adverse event
SD	Standard deviation
FEV_1_	Forced expiratory volume in 1 s
AE	Adverse event
RCT	Randomized controlled trial
BFF	Budesonide/formoterol fumarate
GFF	Glycopyrrolate/formoterol fumarate
SITT	Single-inhaler triple therapy

## References

[1] (2024). Global Strategy for the Diagnosis, Management, and Prevention of Chronic Obstructive Pulmonary Disease: 2024 Report: Global Initiative for Chronic Obstructive Lung Disease (GOLD). https://goldcopd.org/wp-content/uploads/2024/02/GOLD-2024_v1.2-11Jan24_WMV.pdf.

[2] Wang Z., Lin J., Liang L., Huang F., Yao X., Peng K., Gao Y., Zheng J. (2025). Global, regional, and national burden of chronic obstructive pulmonary disease and its attributable risk factors from 1990 to 2021: An analysis for the Global Burden of Disease Study 2021. Respir. Res..

[3] Daniel R.A., Aggarwal P., Kalaivani M., Gupta S.K. (2021). Prevalence of chronic obstructive pulmonary disease in India: A systematic review and meta-analysis. Lung India.

[4] Salvi S., Ghorpade D. (2021). What is the true burden of chronic obstructive pulmonary disease in India and what are its implications at a national level?. Lung India.

[5] Oba Y., Keeney E., Ghatehorde N., Dias S. (2018). Dual combination therapy versus long-acting bronchodilators alone for chronic obstructive pulmonary disease (COPD): A systematic review and network meta-analysis. Cochrane Database Syst. Rev..

[6] Abdool-Gaffar M.S., Calligaro G., Wong M.L., Smith C., Lalloo U.G., Koegelenberg C.F.N., Dheda K., Allwood B.W., Goolam-Mahomed A., van Zyl-Smit R.N. (2019). Management of chronic obstructive pulmonary disease-A position statement of the South African Thoracic Society: 2019 update. J. Thorac. Dis..

[7] Wedzicha J.A., Decramer M., Ficker J.H., Niewoehner D.E., Sandström T., Taylor A.F., D’Andrea P., Arrasate C., Chen H., Banerji D. (2013). Analysis of chronic obstructive pulmonary disease exacerbations with the dual bronchodilator QVA149 compared with glycopyrronium and tiotropium (SPARK): A randomised, double-blind, parallel-group study. Lancet Respir. Med..

[8] Welte T., Miravitlles M., Hernandez P., Eriksson G., Peterson S., Polanowski T., Kessler R. (2009). Efficacy and tolerability of budesonide/formoterol added to tiotropium in patients with chronic obstructive pulmonary disease. Am. J. Respir. Crit. Care Med..

[9] Papi A., Vestbo J., Fabbri L., Corradi M., Prunier H., Cohuet G., Guasconi A., Montagna I., Vezzoli S., Petruzzelli S. (2018). Extrafine inhaled triple therapy versus dual bronchodilator therapy in chronic obstructive pulmonary disease (TRIBUTE): A double-blind, parallel group, randomised controlled trial. Lancet.

[10] Bardsley S., Criner G.J., Halpin D.M.G., Han M.K., Hanania N.A., Hill D., Lange P., Lipson D.A., Martinez F.J., Midwinter D. (2022). Single-inhaler triple therapy fluticasone furoate/umeclidinium/vilanterol versus dual therapy in current and former smokers with COPD: IMPACT trial post hoc analysis. Respir. Med.

[11] Halpin D.M.G., Rothnie K.J., Banks V., Czira A., Compton C., Wood R., Tritton T., Massey O., Wild R., Snowise N. (2022). Comparative Adherence and Persistence of Single- and Multiple-Inhaler Triple Therapies Among Patients with Chronic Obstructive Pulmonary Disease in an English Real-World Primary Care Setting. Int. J. Chronic Obstr. Pulm. Dis..

[12] Beeh K.M., Kuna P., Corradi M., Viaud I., Guasconi A., Georges G. (2021). Comparison of Dry-Powder Inhaler and Pressurized Metered-Dose Inhaler Formulations of Extrafine Beclomethasone Dipropionate/Formoterol Fumarate/Glycopyrronium in Patients with COPD: The TRI-D Randomized Controlled Trial. Int. J. Chronic Obstr. Pulm. Dis..

[13] Wedzicha J.A.E.C.-C., Miravitlles M., Hurst J.R., Calverley P.M., Albert R.K., Anzueto A., Criner G.J., Papi A., Rabe K.F., Rigau D. (2017). Management of COPD exacerbations: A European Respiratory Society/American Thoracic Society guideline. Eur. Respir. J..

[14] Ferguson G.T., Rabe K.F., Martinez F.J., Fabbri L.M., Wang C., Ichinose M., Bourne E., Ballal S., Darken P., DeAngelis K. (2018). Triple therapy with budesonide/glycopyrrolate/formoterol fumarate with co-suspension delivery technology versus dual therapies in chronic obstructive pulmonary disease (KRONOS): A double-blind, parallel-group, multicentre, phase 3 randomised controlled trial. Lancet Respir. Med..

[15] Rabe K.F., Martinez F.J., Ferguson G.T., Wang C., Singh D., Wedzicha J.A., Trivedi R., St Rose E., Ballal S., McLaren J. (2020). Triple Inhaled Therapy at Two Glucocorticoid Doses in Moderate-to-Very-Severe COPD. N. Engl. J. Med..

[16] Lipson D.A., Barnhart F., Brealey N., Brooks J., Criner G.J., Day N.C., Dransfield M.T., Halpin D.M.G., Han M.K., Jones C.E. (2018). Once-Daily Single-Inhaler Triple versus Dual Therapy in Patients with COPD. N. Engl. J. Med..

[17] Vestbo J., Papi A., Corradi M., Blazhko V., Montagna I., Francisco C., Cohuet G., Vezzoli S., Scuri M., Singh D. (2017). Single inhaler extrafine triple therapy versus long-acting muscarinic antagonist therapy for chronic obstructive pulmonary disease (TRINITY): A double-blind, parallel group, randomised controlled trial. Lancet.

[18] Halpin D.M.G. (2023). Clinical Management of COPD in the Real World: Can Studies Reveal Errors in Management and Pathways to Improve Patient Care?. Pragmatic Obs. Res..

[19] Muro S., Kawayama T., Sugiura H., Seki M., Duncan E.A., Bowen K., Marshall J., Megally A., Patel M. (2024). Benefits of budesonide/glycopyrronium/formoterol fumarate dihydrate on lung function and exacerbations of COPD: A post-hoc analysis of the KRONOS study by blood eosinophil level and exacerbation history. Respir. Res..

[20] Singh D., Rabe K.F., Martinez F.J., Krüll M., Jenkins M., Patel M., Dorinsky P. (2022). Relationship between prior inhaled corticosteroid use and benefits ofbudesonide/glycopyrronium/formoterol fumarate dihydrate on exacerbations, symptoms, health-related quality of life, and lung function in patients with chronic obstructive pulmonary disease: Analyses from the ETHOS study. Respir. Med..

[21] Singh D., Martinez F.J., Hurst J.R., Han M.K., Gale C.P., Fredriksson M., Kisielewicz D., Mushunje A., Movitz C., Ojili N. (2025). Effect of Triple Therapy on Cardiovascular and Severe Cardiopulmonary Events in Chronic Obstructive Pulmonary Disease: A Post Hoc Analysis of a Randomized, Double-Blind, Phase 3 Clinical Trial (ETHOS). Am. J. Respir. Crit. Care Med..

[22] Zheng J., Baldi S., Zhao L., Li H., Lee K.H., Singh D., Papi A., Grapin F., Guasconi A., Georges G. (2021). Efficacy and safety of single-inhaler extrafine triple therapy versus inhaled corticosteroid plus long-acting beta2 agonist in eastern Asian patients with COPD: The TRIVERSYTI randomised controlled trial. Respir. Res..

[23] Müllerová H., Chan J.S.K., Heatley H., Carter V., Townend J., Skinner D., Franzén S., Marshall J., Price D. (2024). Budesonide/Glycopyrrolate/Formoterol for the Management of COPD in a UK Primary Care Population: Real-World Use and Early Medication Success. Int. J. Chronic Obstr. Pulm. Dis..

[24] Strange C., Tkacz J., Schinkel J., Lewing B., Agatep B., Swisher S., Patel S., Edwards D., Touchette D.R., Portillo E. (2023). Exacerbations and Real-World Outcomes After Single-Inhaler Triple Therapy of Budesonide/Glycopyrrolate/Formoterol Fumarate, Among Patients with COPD: Results from the EROS (US) Study. Int. J. Chronic Obstr. Pulm. Dis..

[25] Pollack M., Rapsomaniki E., Anzueto A., Rhodes K., Hawkins N.M., Vogelmeier C.F., Marshall J., Müllerová H. (2025). Effectiveness of Single Versus Multiple Inhaler Triple Therapy on Mortality and Cardiopulmonary Risk Reduction in COPD: The SKOPOS-MAZI Study. Am. J. Med..

[26] Calderón-Montero A., de Miguel Diez J., de Simón Gutiérrez R., Campos Téllez S., Chacón Moreno A.D., Alonso Avilés R., González Alonso N., Montero Solís A., Escribano Pardo D. (2025). Triple inhaled therapy of formoterol/glycopyrrolate/budesonide reduces the use of oral corticosteroids and antibiotics during COPD exacerbations in real-world conditions. Semergen.

[27] Brocklebank D., Ram F., Wright J., Barry P., Cates C., Davies L., Douglas G., Muers M., Smith D., White J. (2001). Comparison of the effectiveness of inhaler devices in asthma and chronic obstructive airways disease: A systematic review of the literature. Health Technol. Assess..

