# Supplementary material for: Effectiveness and Safety of Glycopyrronium–Formoterol–Budesonide Triple Therapy in Chronic Obstructive Pulmonary Disease (AIR-FORCE): An Open-Label Multi-Centric Phase 4 Study
Source: Adv Respir Med. 2025 Nov 25;93(6):53. doi: 10.3390/arm93060053 (PMC12729428; doi:10.3390/arm93060053)
Supplement: Supplementary file 1 [file arm-93-00053-s001.zip › arm-3906415-supplementary.pdf]

Supplementary Material

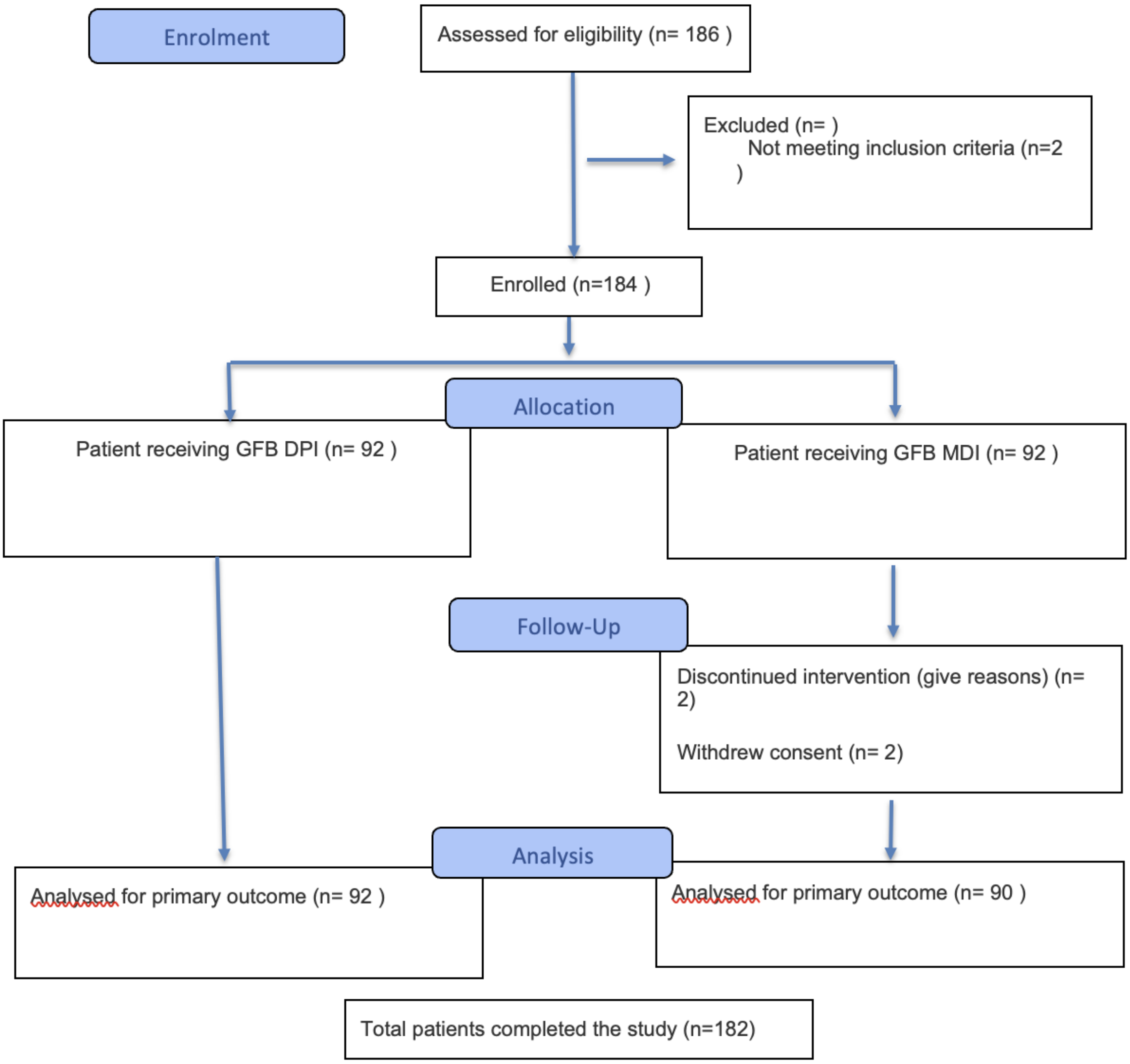

Supplementary Figure 1: *Flowchart for patient selection (CONSORT 2025)*

Note: GFB=glycopyrronium/formoterol fumarate/budesonide; DPI=dry powder inhaler; MDI=metered dose inhaler

Parameter	GFB DPI (N=92) (mean ± SD)	GFB MDI (N=92) (mean ± SD)	Overall (N=184) (mean ± SD)
**Trough FEV_1 in ml**			
Baseline	1156.41±358.81	1200.38±381.33	1178.39±369.89
24 weeks	1623.03±435.07	1734.11±556.49	1677.96±500.54
Change from baseline	466.62±76.26	533.73±175.16	499.57±130.65
P value vs baseline	<0.001	<0.001	<0.001
**Trough FEV_1 %**			
Baseline	49.89±11.11	50.13±11.77	50.00±11.41
24 weeks	66.72±8.73	68.43±9.05	67.57±8.91
Change from baseline	16.83±2.38	18.3±2.72	17.57±2.5
P value vs baseline	<0.001	<0.001	<0.001
**Trough FVC in ml**			

Baseline	1992.08±557.76	2103.04±535.16	2047.56±547.92
24 weeks	2481.21±622.13	2598.89±657.45	2539.40±640.79
Change from baseline	489.13±64.37	495.85±122.29	491.84±92.87
P value vs baseline	<0.001	<0.001	<0.001
**mMRC score**			
Baseline	2.98±0.61	3.05±0.72	3.01±0.66
24 weeks	1.53±0.74	1.61±0.75	1.57±0.75
Change from baseline	-1.45±0.13	-1.44±0.03	-1.44±0.09
P value vs baseline	<0.001	<0.001	<0.001

Supplementary Table 1: *Effectiveness after 24 weeks of GFB (glycopyrronium/formoterol fumarate/budesonide) triple therapy given as dry powder inhaler (DPI) or metered dose inhaler (MDI) formulation, and overall effectiveness, compared to baseline*

Note: FEV_1=forced expiratory volume in 1 second; FVC=forced vital capacity; mMRC=modified medical research council; GFB=glycopyrronium/formoterol fumarate/budesonide; DPI=dry powder inhaler; MDI=metered dose inhaler

Visit	N	GFB DPI n (%)	GFB MDI n (%)	Overall n (%)
Week 4				
Mild	184	3 (3.26%)	2 (2.17%)	5 (2.72%)
Moderate	-			
Severe	-			
Week 12				
Mild	184	2 (2.17%)	2 (2.17%)	4 (2.17%)
Moderate	-			
Severe	-			
Week 24				
Mild	182	2 (2.17%)	1 (1.12%)	3 (1.65%)
Moderate	-			
Severe	-			

Supplementary Table 2: *Number of subjects with exacerbations (mild/ moderate/ severe) over 24 weeks*

Note: GFB=glycopyrronium/formoterol fumarate/budesonide; DPI=dry powder inhaler; MDI=metered dose inhaler

Study Duration	Rescue Medication Used	GFB DPI n (%)	GFB MDI n (%)	Overall n (%)
Week 0-4	Salbutamol	8 (8.70%)	7 (7.60%)	15 (8.15%)
Week 4-12	Salbutamol	6 (6.52%)	5 (5.43%)	11 (5.98%)
Week 12-24	Salbutamol	3 (3.34%)	3 (3.34%)	6 (3.29%)

Supplementary Table 3: *Rescue medication use over 24 weeks of therapy with GFB*

Note: GFB=glycopyrronium/formoterol fumarate/budesonide; DPI=dry powder inhaler; MDI=metered dose inhaler

Visit	GFB DPI Compliance	N	GFB MDI Compliance	N	Overall Compliance	N
Week 4	100%	92	100%	92	100%	184
Week 12	100%	92	100%	92	100%	184
Week 24	100%	92	100%	90	100%	182

Supplementary Table 4: *Compliance with study medication over 24 weeks of therapy*

Note: GFB=glycopyrronium/formoterol fumarate/budesonide; DPI=dry powder inhaler; MDI=metered dose inhaler

Visit	GFB DPI		GFB MDI		Overall	
	Yes, n (%)	*No, n (%)*	*Yes, n (%)*	*No, n (%)*	*Yes, n (%)*	*No, n (%)*
Q1. Do you find it easy to fit your COPD medication into your everyday life?						
**Week 4**	90 (97.83%)	2 (2.17%)	90 (97.83%)	2 (2.17%)	180 (97.83%)	4 (2.17%)
**Week 12**	92 (100%)	-	92(100%)	-	184(100%)	-
**Week 24**	92(100%)	-	90(100%)	-	182(100%)	-
Q2. When you take your medication, do you feel confident that your COPD symptoms will be controlled?						
**Week 4**	89 (96.74%)	3 (3.26%)	85 (92.39%)	7 (7.61%)	174 (94.57%)	10 (5.43%)
**Week 12**	91 (98.91%)	1 (1.09%)	88 (95.65%)	4 (4.35%)	179 (97.28%)	5 (2.72%)
**Week 24**	91 (98.91%)	1 (1.09%)	88 (97.78%)	2 (2.23%)	179 (98.35%)	3 (1.65%)
Q3. Do you worry that you are not taking the right medication for your COPD symptoms?						
**Week 4**	6 (6.52%)	86 (93.48%)	8 (8.70%)	84 (91.30%)	14 (7.61%)	170 (92.39%)
**Week 12**	1 (1.09%)	91 (98.91%)	7 (7.61%)	85 (92.39%)	8 (4.35%)	176 (95.65%)
**Week 24**	1 (1.09%)	91 (98.91%)	3 (3.26%)	87 (94.57%))	4 (2.20%)	178 (97.80%)
Q4. Do you find it easy to carry your COPD inhaler(s) with you, and do not have any taste or smell complaints?						
**Week 4**	91 (98.91%)	1 (1.09%)	90 (97.83%)	2 (2.17%)	181 (98.37%)	3 (1.63%)
**Week 12**	92 (100%)	-	92 (100%)	-	184 (100%)	-
**Week 24**	92 (100%)	-	90 (100%)	-	182 (100%)	-
Q5. Do you feel confident in using your inhaler(s)?						
**Week 4**	91 (98.91%)	1 (1.09%)	91 (98.91%)	1 (1.09%)	182 (98.91%)	2 (1.09%)
**Week 12**	92 (100%)	-	90 (97.83%)	2 (2.17%)	182 (98.91%)	2 (1.09%)
**Week 24**	92 (100%)	-	88 (97.78%)	2 (2.23%)	180 (98.90%)	2 (1.10%)

Supplementary Table 5: *Patient satisfaction with GFB treatment over 24 weeks for GFB-MDI, GFB-DPI, and overall groups*

Note: GFB=glycopyrronium/formoterol fumarate/budesonide; DPI=dry powder inhaler; MDI=metered dose inhaler

Visit	GFB DPI		GFB MDI		Overall	
	Yes, n (%)	*No, n (%)*	*Yes, n (%)*	*No, n (%)*	*Yes, n (%)*	*No, n (%)*
Q1. Do you feel that the patient's COPD medication fits easily into their everyday life?						
**Week 4**	89 (96.74%)	3 (3.26%)	91 (98.91%)	1 (1.09%)	180 (97.82%)	4 (2.17%)
**Week 12**	90 (97.83%)	2 (2.17%)	92 (100%)	-	182 (98.91%)	2 (1.09%)
**Week 24**	92 (100%)	-	90 (100%)	-	182 (100%)	-
Q2. Do you feel confident in the ability of this medication to control patients COPD Symptoms?						
**Week 4**	90 (97.83%)	2 (2.17%)	91 (98.91%)	1 (1.09%)	181 (98.37%)	3 (1.63%)
**Week 12**	92 (100%)	-	90 (97.83%)	2 (2.17%)	182 (98.91%)	2 (1.09%)
**Week 24**	92 (100%)	-	90 (100%)	-	182 (100%)	-
Q3. Do you have any worries or uncertainties about whether this is the appropriate medication for this patient?						
**Week 4**	3 (3.26%)	89 (96.74%)	6 (6.52%)	86 (93.48%)	9 (4.89%)	175 (95.11%)
**Week 12**	3 (3.26%)	89 (96.74%)	3 (3.26%)	89 (96.74%)	6 (3.26%)	178 (96.74%)
**Week 24**	-	92 (100%)	-	90 (100%)	-	182 (100%)
Q4. Do you feel it is convenient for the patient to carry their COPD inhaler with them and not have any taste or smell complaints?						
**Week 4**	91 (98.91%)	1 (1.09%)	91 (98.91%)	1 (1.09%)	182 (98.91%)	2 (1.09%)
**Week 12**	92 (100%)	-	92 (100%)	-	184 (100%)	-
**Week 24**	92 (100%)	-	90 (100%)	-	182 (100%)	-
Q5. Does the patient demonstrate confidence in their ability to use their inhaler(s) effectively?						
**Week 4**	92 (100%)	-	91 (98.91%)	1 (1.09%)	183 (99.46%)	1 (0.54%)
**Week 12**	92 (100%)	-	91 (98.91%)	1 (1.09%)	183 (99.46%)	1 (0.54%)
**Week 24**	92 (100%)	-	89 (98.89%)	1 (1.12%)	181 (99.45%)	1 (0.54%)

Supplementary Table 6*: Physician satisfaction with GFB treatment over 24 weeks for GFB-MDI, GFB-DPI, and overall groups*

Note: GFB=glycopyrronium/formoterol fumarate/budesonide; DPI=dry powder inhaler; MDI=metered dose inhaler

Adverse Event	Number of adverse events reported, n (%)		
	GFB DPI (N=92)	GFB MDI (N=92)	Overall (N=184)
All TEAEs	16 (17.39%)	13 (14.13%)	29 (15.76%)
Respiratory, thoracic and mediastinal disorders			
Cough	7 (7.60%)	3 (3.26%)	10 (5.43%)
URTI	-	4 (4.34%)	4 (2.17%)
Cold	1 (1.09%)	-	1 (0.54%)
Infections and Infestations			
UTI	2 (2.17%)	-	2 (1.09%)
Musculoskeletal & Connective Tissue disorder			
Muscle spasm	-	2 (2.17%)	2 (1.09%)
Back pain	1 (1.09%)	-	1 (0.54%)
Nervous System Disorder			
Headache	-	2 (2.17%)	2 (1.09%)
General disorder & administration site conditions			
Fever	2 (2.17%)	-	2 (1.09%)
Gastrointestinal disorders			
Dry mouth	1 (1.09%)	-	1 (0.54%)
Diarrhoea	-	1 (1.09%)	1 (0.54%)
Nausea	1 (1.09%)	-	1 (0.54%)
Heartburn	1 (1.09%)	-	1 (0.54%)
Renal and Urinary Disorders			
Burning micturition	-	1 (1.09%)	1 (0.54%)

Supplementary Table 7: *Detailed group-wise safety outcomes of 24 weeks of GFB therapy for COPD management*

Note: TEAE=treatment-emergent adverse event; URTI=upper respiratory tract infections; UTI=urinary tract infections; GFB=glycopyrronium/formoterol fumarate/budesonide; DPI=dry powder inhaler; MDI=metered dose inhaler

Supplementary Table 8.

Section/topic	No	CONSORT 2025 checklist item description	Reported on page no.
**Title and abstract**			
Title and structured abstract	1a	Identification as a randomised trial	1
	1b	Structured summary of the trial design, methods, results, and conclusions	1
**Open science**			
Trial registration	2	Name of trial registry, identifying number (with URL) and date of registration	11
Protocol and statistical analysis plan	3	Where the trial protocol and statistical analysis plan can be accessed	NA
Data sharing	4	Where and how the individual de-identified participant data (including data dictionary), statistical code and any other materials can be accessed	NA
Funding and conflicts of interest	5a	Sources of funding and other support (eg, supply of drugs), and role of funders in the design, conduct, analysis and reporting of the trial	11
	5b	Financial and other conflicts of interest of the manuscript authors	11
**Introduction**			
Background and rationale	6	Scientific background and rationale	2
Objectives	7	Specific objectives related to benefits and harms	3
**Methods**			**3**
Patient and public involvement	8	Details of patient or public involvement in the design, conduct and reporting of the trial	3
Trial design	9	Description of trial design including type of trial (eg, parallel group, crossover), allocation ratio, and framework (eg, superiority, equivalence, non-inferiority, exploratory)	3
Changes to trial protocol	10	Important changes to the trial after it commenced including any outcomes or analyses that were not prespecified, with reason	NA
Trial setting	11	Settings (eg, community, hospital) and locations (eg, countries, sites) where the trial was conducted	3, Suppl Table 1
Eligibility criteria	12a	Eligibility criteria for participants	3
	12b	If applicable, eligibility criteria for sites and for individuals delivering the interventions (eg, surgeons, physiotherapists)	NA
Intervention and comparator	13	Intervention and comparator with sufficient details to allow replication. If relevant, where additional materials describing the intervention and comparator (eg, intervention manual) can be accessed	3
Outcomes	14	Prespecified primary and secondary outcomes, including the specific measurement variable (eg, systolic blood pressure), analysis metric (eg, change from baseline, final value, time to event), method of aggregation (eg, median, proportion), and time point for each outcome	4
Harms	15	How harms were defined and assessed (eg, systematically, non-systematically)	3, 4
Sample size	16a	How sample size was determined, including all assumptions supporting the sample size calculation	

	16b	Explanation of any interim analyses and stopping guidelines	NA
Randomisation:			NA
Sequence generation	17a	Who generated the random allocation sequence and the method used	
	17b	Type of randomisation and details of any restriction (eg, stratification, blocking and block size)	NA
			**Reported on page no.**
Allocation concealment mechanism	18	Mechanism used to implement the random allocation sequence (eg, central computer/telephone; sequentially numbered, opaque, sealed containers), describing any steps to conceal the sequence until interventions were assigned	NA
Implementation	19	Whether the personnel who enrolled and those who assigned participants to the interventions had access to the random allocation sequence	NA
Blinding	20a	Who was blinded after assignment to interventions (eg, participants, care providers, outcome assessors, data analysts)	NA
	20b	If blinded, how blinding was achieved and description of the similarity of interventions	NA
Statistical methods	21a	Statistical methods used to compare groups for primary and secondary outcomes, including harms	4
	21b	Definition of who is included in each analysis (eg, all randomised participants), and in which group	NA
	21c	How missing data were handled in the analysis	NA
	21d	Methods for any additional analyses (eg, subgroup and sensitivity analyses), distinguishing prespecified from post hoc	NA
**Results**			
Participant flow, including flow diagram	22a	For each group, the numbers of participants who were randomly assigned, received intended intervention, and were analysed for the primary outcome	4
	22b	For each group, losses and exclusions after randomisation, together with reasons	4
Recruitment	23a	Dates defining the periods of recruitment and follow-up for outcomes of benefits and harms	4
	23b	If relevant, why the trial ended or was stopped	NA
Intervention and comparator delivery	24a	Intervention and comparator as they were actually administered (eg, where appropriate, who delivered the intervention/comparator, how participants adhered, whether they were delivered as intended (fidelity))	4
	24b	Concomitant care received during the trial for each group	NA
Baseline data	25	A table showing baseline demographic and clinical characteristics for each group	4-5
Numbers analysed, outcomes and estimation	26	For each primary and secondary outcome, by group:<br>● the number of participants included in the analysis<br>● the number of participants with available data at the outcome time point<br>● result for each group, and the estimated effect size and its precision (such as 95% confidence interval)<br>● for binary outcomes, presentation of both absolute and relative effect size	5-8
Harms	27	All harms or unintended events in each group	7
Ancillary analyses	28	Any other analyses performed, including subgroup and sensitivity analyses, distinguishing pre-specified from post hoc	NA
**Discussion**			**8**
Interpretation	29	Interpretation consistent with results, balancing benefits and harms, and considering other relevant evidence	8, 9

Limitations	30	Trial limitations, addressing sources of potential bias, imprecision, generalisability, and, if relevant, multiplicity of analyses	10
